# Angiotensin receptor-neprilysin inhibitors and cardiac remodeling

**DOI:** 10.1590/1414-431X2023e12616

**Published:** 2023-04-07

**Authors:** A.S. Ryazanov, E.V. Shikh, M.V. Makarovskaya, A.A. Kudryavtsev

**Affiliations:** 1 I.M. Sechenov First Moscow State Medical University, Ministry of Health of Russia (Sechenov University), Moscow, Russia; 2 Clinical and Diagnostic Center No. 4, Moscow Department of Health, Moscow, Russia

**Keywords:** Heart failure, Cardiac remodeling, Neprilysin, Angiotensin receptor blockers

## Abstract

The aim of this study was to determine how sacubitril/valsartan compared with valsartan in an outpatient setting affects left ventricular remodeling in heart failure with reduced ejection fraction and functional (or secondary) mitral regurgitation (SMR) due to the effect of dual inhibition of the renin-angiotensin system and neprilysin. The outpatient study included 90 patients with chronic SMR who were followed up for 12 months. They received sacubitril/valsartan or valsartan instead of the more commonly used angiotensin-converting enzyme inhibitor enalapril for heart failure, in addition to standard medical therapy for heart failure. The difference in NT-proBNP change between groups was the primary endpoint. Changes in effective regurgitation orifice area, left ventricular ejection fraction, left ventricular end-systolic and end-diastolic volume indices, left atrial volume index, E/e' index, and exercise tolerance on the 6-minute walk test were secondary endpoints. In the treatment efficacy analysis, NT-proBNP levels decreased significantly by 37% in the sacubitril/valsartan group and by 11% in the valsartan group (P<0.001). Ejection fraction and exercise tolerance (increase in walking distance in the 6-min test) increased in the sacubitril/valsartan group (P<0.05). Also, in this group, the effective area of the regurgitation orifice, the left atrial volume index, the E/e' index, and the indices of the end-systolic and end-diastolic volume of the left ventricle (P<0.05) decreased more pronouncedly. Compared with valsartan, treatment with sacubitril/valsartan led to a significant improvement in cardiac remodeling in patients with SMR and heart failure with reduced ejection fraction.

## Introduction

Heart failure (HF) is a multisystemic disease characterized by significant disturbances in cardiac physiology and a multitude of myocardial structural and functional changes (1). HF remains a global problem and is associated with high morbidity, mortality, and treatment costs (including readmissions and outpatient visits) (2). The Framingham Heart Study has shown that the prevalence of chronic heart failure (CHF) in the population continues to increase. In Russia, according to the EPOCHA-CHF and EPOCHA-O-CHF studies, CHF is observed in 7% of the general population (7.9 million people). Clinically manifested CHF (II-IV functional class) is observed in 4.5% of the population (5.1 million people) (3,4). HF is very often a comorbidity: more than 80% of patients have more than 2-3 concomitant diseases (5).

Common diseases such as hypertension, coronary heart disease, diabetes mellitus, chronic kidney disease, and chronic heart failure may be accompanied by functional (or secondary) mitral regurgitation (SMR). Valvular heart disease accounts for 7-8% of HF cases. SMR occurs when normal or near-normal mitral leaflets fail to close adequately due to underlying left ventricular (LV) dysfunction or dilatation of the mitral annulus. SMR often occurs in CHF with reduced left ventricular ejection fraction as a result of LV remodeling that prevents coaptation of the valve leaflets. SMR has a worse prognosis than primary mitral regurgitation (MR). Despite the great diversity and improvements of drug therapy and surgical interventions, SMR continues to progress in most cases. This leads to further remodeling of the left ventricular myocardium and deterioration of the HF functional class in patients. The binary approach to MR, being either a primary valvular problem or valvular regurgitation secondary to ventricular dysfunction, should be considered, which may have therapeutic value. Drug therapy is limited for primary MR (1).

The results of the COAPT study (a randomized trial of transcatheter mitral valve leaflet approximation in patients with heart failure and secondary mitral regurgitation) have revived enthusiasm for transcatheter mitral valve repair in SMR (6). Patients with heart failure and symptomatic moderate-to-severe MR have been shown to have a significant reduction in hospital admissions for heart failure and deaths after percutaneous edge-to-edge mitral valve repair compared with patients receiving only optimal drug therapy.

The Mitra-FR trial (Percutaneous repair with the mitraclip device for severe functional/secondary mitral regurgitation) (7) is another very important study that is more complementary than competitive. The COAPT trial indicates that a highly select group of patients with moderate-severe secondary MR without excessive ventricular dilatation with symptomatic CHF could benefit from the MitraClip despite maximized guideline-directed medical therapy. In addition, the Mitra-FR trial indicates that patients with less severe MR and excessive ventricular dilatation do not benefit from the MitraClip (8). In patients with greater volumetric MR and less dilated ventricles as in the COAPT trial, it is plausible that the correction of MR via a MitraClip would result in a greater improvement in CHF symptoms than in patients like those in the Mitra-FR trial with severely dilated ventricles. The comparison in the COAPT trial was between MitraClip and palliative therapy. In contrast, continued optimization of medical therapy was available for patients in both groups after randomization in the Mitra-FR trial.

Guideline-based medical therapy is important because MR is dynamic and changes in response to medications, loading conditions, and hemodynamics.

These studies highlight the importance of aggressive drug therapy for heart failure with reduced ejection fraction (HFrEF) and open the door to targeted therapy in patients with a significant burden of SMR that can contribute to left ventricular systolic dysfunction and volume overload. Effective drug therapy reverses LV remodeling and decreases the degree of mitral regurgitation in SMR (9).

In the case of SMR and HF, the treatment of choice includes beta-blockers, angiotensin-converting enzyme (ACE) inhibitors, angiotensin receptor blockers (ARBs), and mineralocorticoid receptor antagonists (MRA), which can partially attenuate LV dilatation and remodeling after myocardial injury. Recently, sacubitril, a new combination of valsartan and neprilysin inhibitor, which has more pronounced hemodynamic and neurohormonal effects, has been used as a replacement for ACE inhibitors or ARBs in HFrEF patients (9).

The main objective of our study was to verify whether sacubitril/valsartan is better at reversing LV remodeling in HFrEF with SMR than valsartan alone in an outpatient setting because of the dual inhibition of the renin-angiotensin system and neprilysin.

## Material and Methods

### Study design

A single-center, controlled, prospective, non-randomized outpatient study was performed.

### Inclusion criteria

Screening inclusion criteria included age ≥40 years, stable HF with New York Heart Association (NYHA) class II or III symptoms, EF 35 to 40%, and duration of significant SMR >6 months, which were assessed using transthoracic echocardiography (EchoCG). Significant SMR has the following criteria: normal mitral valve leaflets and chords, regional or global LV wall motion abnormalities with leaflet fixation, and MR with an effective regurgitation orifice area of >0.1 cm^2^ lasting more than 6 months despite treatment with beta-blockers and ACE inhibitors (or ARBs). Patients were required to be on a stable dose of beta-blockers and ACE inhibitors (or ARBs) for at least 4 weeks prior to screening (but there was a mandatory withdrawal of ACE inhibitors (or ARBs) at least 36 h before the appointment for sacubitril/valsartan medication).

### Exclusion criteria

Screening exclusion criteria included systolic blood pressure <100 mmHg, glomerular filtration rate <30 mL·min·1.73 m^2^, serum potassium level >5.0 mmol/L, or a history of angioedema. Patients were also excluded from the study if they had any evidence of structural mitral valve disease, NYHA class IV symptoms, previous valvular intervention, hospitalization within the previous 6 weeks, a history of acute coronary syndrome, stroke, cardiovascular surgery or percutaneous coronary intervention within 3 months, significant myocardial ischemia requiring coronary revascularization, planned coronary revascularization, and mitral valve intervention.

### Terms and conditions

The study was conducted at the Clinical Diagnostic Center No. 4 (Russia) from December 2018 to December 2019, inclusive.

### Ethical review

The study protocol was approved by the Joint Ethics Committee (protocol number 100/18, December 10, 2018). All necessary permits were received. Written informed consent was obtained from all participants prior to enrollment in the study. The study was conducted in accordance with the principles of Good Clinical Practice and the Declaration of Helsinki.

### Description of the medical intervention

The sample size was determined using an online statistical processing calculator for medical research data. Two groups matched for sex, age, and disease stage were recruited. The present study patients were divided into two groups: 46 patients took valsartan and 44 received sacubitril/valsartan (36 h after the last dose of ACE inhibitors). Patients started taking valsartan at 40 to 80 mg twice daily or sacubitril/valsartan at 24/26 to 49/51 mg twice daily. The dose was titrated based on tolerability at 4-week intervals up to the maximum dose of 160 mg twice daily for valsartan or 97/103 mg twice daily for sacubitril/valsartan. All other medications (beta-blockers, diuretics, aldosterone antagonists, and digitalis) were continued. Patients were treated for 12 months.

### Study outcomes

The primary endpoint was the between-group difference in NT-proBNP change over 12 months of follow-up.

The secondary endpoints included between-group differences in change in the effective regurgitation orifice area in SMR, left ventricular ejection fraction, left ventricular end-systolic and end-diastolic volume indices, left atrial volume index, E/e' index, and exercise tolerance (6-min walk test) at 12 months of follow-up.

### Study procedures

The cardiac biomarker NT-proBNP was analyzed in the laboratory using stored samples collected prior to administration of the study medicines and after 12 months of treatment. The concentration of NT-proBNP in blood serum was determined by solid-phase chemiluminescence enzyme immunoassay (sandwich assay) using IMMULITE 2000 analyzer with commercial NT-proBNP kits (IMMULITE, Siemens, USA).

EchoCG is the gold standard in the diagnosis of SMR in HFrEF. Echocardiographic evaluation was performed at baseline and at the 12-month follow-up visit. EchoCG was performed in standard projections using ultrasonic devices Logiq 500 and Vivid 3 Expert (GE, USA) in greyscale, M- and B-modes. Color, continuous, and pulsed wave Doppler methods were used for quantitative and qualitative assessment of functional and organic changes in the heart and great vessels during standard EchoCG protocol. Severity of SMR should be assessed by one and the same experienced echocardiographer using an integrated multi-parametric approach. Comprehensive Doppler-echocardiographic examinations were performed at enrollment and at follow-up. The echocardiographic measurements were performed by an independent investigator who was blinded to the study group.

End-diastolic and end-systolic volume of the left ventricle was measured on an apical 4-chamber image. End-systolic volume, end-diastolic volume, and ejection fraction of the left ventricular was calculated by the biplane Simpson method.

The quantitative assessment is highly operator-dependent with limited reproducibility, inaccurate in the presence of an elliptical regurgitant orifice (observed frequently in SMR) or multiple jets, and often overlooked in everyday clinical practice. To mitigate the risk of error, multiple parameters should be assessed (vena contracta, pulmonary vein systolic flow reversal, proximal isovelocity surface area (PISA) radius, and the subsequently derived EROA and regurgitant volume) (10).

The effective regurgitation orifice area was determined by dividing the regurgitation flow rate, calculated as 2πr^2^ × Nyquist limit, where r is the radius of the proximal isovelocity surface area, by the Nyquist limit, the peak regurgitation flow rate. The radius of the proximal isovelocity surface area was measured at early, middle, and late systoles with the most satisfactory proximal isovelocity surface area for the hemisphere and averaged. A significant change in the severity of mitral regurgitation was previously determined as the absolute value of the change in the effective area of the orifice regurgitation >0.1 cm^2^ or a >50% change in the effective area of the regurgitation orifice compared with baseline. The volume of regurgitation was estimated as the effective area of the orifice of regurgitation multiplied by the integral of the velocity from the mitral regurgitation jet. According to mitral valve hemodynamic parameters, patients were considered at risk of mitral regurgitation in the absence of a MR jet or the presence of a small jet in the central region in the Doppler mode, with a small isthmus of regurgitation (vena contracta <0.30 cm); with progressive MR, with an effective area of the orifice of regurgitation <0.20 cm^2^ and regurgitation volume <30 mL; and with severe MR, with an effective area of the regurgitation opening ≥0.20 cm^2^ and regurgitation volume ≥30 mL.

The E/e' index is defined as the ratio of the maximum early diastolic inflow of the left ventricle E to the maximum rate of early diastolic excursion of the mitral annulus e' (11). The average value of the E/e' index >15 at rest has significant diagnostic value for increased LV filling pressure (12). Mitral valve echocardiogram was analyzed to determine the leaflet coaptation point and peak systolic position of the mitral valve leaflets relative to the atrioventricular plane. Incomplete closure of the mitral valves was determined as the inability of one or both leaflets to reach the atrioventricular plane at the point of its maximum systolic movement from above.

### Statistical analysis

The baseline mean NT-proBNP was assumed to be >125 pg/mL and the dropout rate, 10%. Based on these assumptions, a sample of 90 patients divided into 2 groups would provide 80% power if a two-tailed *t*-test at α=0.05 was used. Quantitative indicators were evaluated for compliance with the normal distribution using the Kolmogorov-Smirnov test. Quantitative indicators with a normal distribution were described using arithmetic means, standard deviations (SD), and upper and lower bounds of the 95% confidence interval (95%CI). Categorical data were described with absolute values and percentages. When comparing normally distributed scores calculated for two related samples, paired Student's *t*-test was used. A P value <0.05 was considered statistically significant for all tests. Statistical analysis was carried out using the StatTech v. 2.5.5 (Stattech LLC., Russia).

## Results

The study included 90 patients; 44 patients received sacubitril/valsartan and 46 received valsartan. The mean age of the patients was 61.5±5.3 years, and 63% were men. The cause of SMR was ischemic in 33 (36.7%) patients and non-ischemic in 57 (63.3%), while atrial fibrillation was present in 25 (27.8%) patients (Table 1). Non-ischemic SMR was due to long-term hypertension (80%) or dilated cardiomyopathy (20%). All patients were taking either an ACE inhibitor or an ARB, and 91% took diuretics prior to enrollment (Table 2). There was a mandatory withdrawal of ACE inhibitors (or ARBs) at least 36 h before the administration of sacubitril/valsartan. All patients from both groups reached the required dose of drugs. Optimal doses and other drugs used as concomitant therapy for CHF were beta-blocker bisoprolol, 5 mg per day; diuretics: hydrochlorothiazide 100 mg or indapamide 5.0 mg per day; aldosterone antagonists: eplerenone, 50 mg once; digitalis: 0.5 mg/day. Mean LV ejection fraction was 37.1±4.2%. The treatment groups were generally well balanced in terms of the baseline clinical and echocardiographic characteristics (Table 3) (13).

**Table 1:** Data of patients included in the study according to treatment.

Characteristics	Valsartan (n=46)	Sacubitril/valsartan (n=44)	P value
Age, years	60.72±4.95	62.30±5.50	0.09
Male, n (%)	33 (71.7)	30 (68.2)	0.46
Height, cm	163.48±9.66	162.75±7.62	0.34
Body mass index, kg/m^2^	23.246±1.417	24.477±1.489	0.03
Arterial hypertension, n (%)	24 (52.2)	24 (54.5)	0.49
Diabetes mellitus, n (%)	15 (32.6)	14 (31.8)	0.88
History data, n (%)			
Hospitalization due to HF	27 (58.7)	26 (59.1)	0.45
Myocardial infarction	9 (19.6)	10 (22.7)	0.51
PCI	10 (22.2)	7 (15.9)	0.27
Stroke	2 (4.3)	4 (9.1)	0.33
Atrial fibrillation, n (%)	12 (26.1)	13 (29.5)	0.15
SBP, mmHg	139.41±8.23	133.70±7.41	0.09
DBP, mmHg	88.50±3.72	85.61±4.58	0.11
Creatinine level, mmol/L	89.43±13.32	91.86±13.34	0.45
Potassium level, mmol/L	4.124±0.241	4.214±0.310	0.53
Smoking, n (%)	10 (21.7)	12 (27.3)	0.36
NYHA FC, n (%)			0.34
II	40 (87.0)	37 (84.1)	
III	6 (13)	7 (15.9)	
Cause of functional MR, n (%)			0.42
Ischemic	16 (34.8)	17 (38.6)	
Non-ischemic	30 (65.2)	27 (61.4)	

Data are reported as means and SD or number and percentage. Student's *t*-test or chi-squared test. HF: heart failure; PCI: percutaneous coronary intervention; SBP: systolic blood pressure; DBP: diastolic blood pressure; NYHA: New York Heart Association; FC: functional class; MR: mitral regurgitation.

**Table 2:** Drug therapy of patients prior to enrollment in the study according to treatment.

Primary therapy, n (%)	Valsartan (n=46)	Sacubitril/valsartan (n=44)	P value
ACE inhibitors	11 (23.9)	16 (36.4)	0.23
ARBs	29 (63.0)	27 (61.4)	0.25
Diuretics	43 (93.5)	39 (88.6)	0.74
Digitalis	10 (21.7)	10 (22.7)	0.15
Beta blockers	40 (87.0)	34 (77.3)	0.88
Aldosterone antagonists	20 (43.5)	15 (34.1)	0.29
Statins	30 (65.2)	23 (52.3)	0.40
Rivaroxaban	12 (26)	13 (30)	0.35
Aspirin Cardio	30 (65)	27 (61)	0.28

ACE: angiotensin-converting enzyme; ARBs: angiotensin receptor blockers. Chi-squared test.

**Table 3:** Echocardiographic data of patients included in the study according to treatment.

Echocardiographic data	Valsartan (n=46)	Sacubitril/valsartan (n=44)	P value
End-systolic size, mm	53.91±6.20	51.32±5.39	0.13
End-diastolic size, mm	61.65±6.25	64.73±4.33	0.22
End-systolic volume, mL	146.39±23.55	138.23±16.74	0.34
Index end-systolic volume, mL/m^2^	82.87±16.70	84.59±14.34	0.11
End-diastolic volume, mL	161.89±49.05	174.09±36.25	0.41
Index end-diastolic volume, mL/m^2^	116.85±21.56	118.16±16.12	0.19
Ejection fraction, %	36.83±2,17	37.39±2,49	0.27
Volume of regurgitation, mL	34.52±6.51	34.36±7.52	0.06
Effective regurgitation orifice area, cm^2^	0.23±0.03	0.22±0.03	0.14
E/e' index	14.76±3.49	16.27±4.48	0.02

Data are reported as means and SD. Student's *t*-test. E: maximum rate of early diastolic filling of the left ventricle; e': maximum speed of the early diastolic movement of the fibrous ring of the mitral valve.

### Primary results of the study

**Figure 1:**
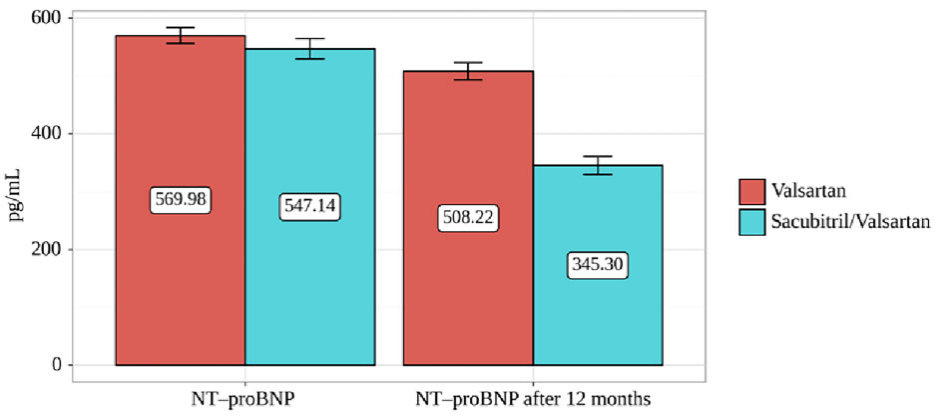
Changes in dynamics depending on treatment method. Data are reported as means and SD. Significant differences were found between groups at baseline (P<0.05) and at the 12-month follow-up (P<0.001) (Student's *t*-test) and within groups at the two time-points (P<0.001) (paired Student's *t*-test).

During the 12 months of follow-up, NT-proBNP level decreased by 37% in the sacubitril/valsartan group and by 11% in the valsartan group. Thus, a statistically significant difference was found between the groups in terms of change from baseline (P<0.001) (Table 4 and Figure 1).

**Table 4:** Changes achieved in the primary endpoint in patients who completed the study.

Treatment method	Stages of observation				P value
	NT-proBNP at baseline, pg/mL		NT-proBNP after 12 months, pg/mL		
	Mean±SD	95%CI	Mean±SD	95%CI	
Valsartan (n=46)	569.98±45.82	556.37-583.59	508.22±50.46	493.23-523.20	<0.001
Sacubitril/valsartan (n=44)	547.14±58.88	529.23-565.04	345.30±50.46	329.95-360.64	<0.001
P	0.042		<0.001		-

Student's *t*-test or paired Student's *t*-test.

### Secondary results of the study

In a treatment efficacy analysis including 90 patients, the effective regurgitation orifice area decreased by 25 and 13% in the sacubitril/valsartan and valsartan groups, respectively (P<0.05) (Table 5).

**Table 5:** Echocardiographic changes in patients who completed the study and changes achieved on the 6-min walk test according to treatment.

Characteristics	Initial data			Results after 12 months			P value
	Valsartan(n=46)	Sacubitril/valsartan(n=44)		Valsartan(n=46)	Sacubitril/valsartan(n=44)		
Effective regurgitation orifice area, cm^2^	0.23±0.03	0.22±0.03		0.20±0.03	0.16±0.03		0.018
Ejection fraction, %	36.83±2.17	37.39±2.49		40.65±3.95	45.61±3.70		0.031
Index end-diastolic volume, mL/m^2^	116.85±21.56	118.16±16.12		111.02±21.69	103.77±14.39		0.03
Index end-systolic volume, mL/m^2^	82.87±16.70	84.59±14.34		76.20±16.43	62.82±10.84		0.026
Left atrial volume index, mL/m^2^	29.46±4.42	29.95±4.78		29.78±3.71	27.70±4.58		0.02
E/e' index	14.76±3.49	16.27±4.48		13.59±3.44	13.68±4.42		0.002
6-min walk test, m	277.54±27.95	297.48±47.57		313.22±37.92	400.52±48.41		0.043

Data are reported as means and SD. E: maximum rate of early diastolic filling of the left ventricle; e': maximum speed of the early diastolic movement of the fibrous ring of the mitral valve. Student's *t*-test.

Compared with participants treated with valsartan, participants treated with sacubitril/valsartan experienced a greater decrease in the index of the LV end-diastolic volume (from 118.16±16.12 to 103.77±14.39 mL/m^2^ for sacubitril/valsartan compared with 116.85±21.56 to 111.02±21.69 mL/m^2^ for valsartan; P=0.004) and LV end-systolic volume index (from 84.59±14.34 to 62.82±10.84 mL/m^2^ with sacubitril/valsartan compared with 82.87±16.70 to 76.20±16.43 mL/m^2^ with valsartan), which led to an increase in LV ejection fraction and stroke volume. This improvement in systolic function and volumetric remodeling was associated with a decrease in visually graded mitral regurgitation valve.

The volume index of the left atrium decreased (from 29.95 to 27.7 mL/m^2^ in the sacubitril/valsartan group compared with 29.46 to 29.78 mL/m^2^ in the valsartan group). On ultrasound from the apical four-chamber position and along the short axis of the heart from the parasternal and subcostal positions after detecting tricuspid regurgitation, color Doppler mapping can accurately measure the velocity of the transtricuspid regurgitation jet. Knowing the maximum rate of regurgitation, using the simplified Bernoulli equation (ΔP=V2), it is possible to calculate the pressure difference between the right ventricle and the right atrium. By adding to this value the value of right atrial pressure corresponding to the central venous pressure, one can determine the systolic pressure in the right ventricle, which, in the absence of pulmonary valve stenosis, corresponds to the systolic pressure in the pulmonary artery. Using this method, we found a tendency for a decrease in systolic pressure in the right ventricle, which could be measured in the availability of tricuspid regurgitation. In addition, diastolic function improved. The E/e' index decreased (by 2.59 for sacubitril/valsartan compared to 1.17 for valsartan) (Table 5).

Ejection fraction increased by 8.2% in the sacubitril/valsartan group and by 3.8% in the valsartan group.

During the follow-up period, an increase in exercise tolerance was noted (distance in the 6-min walk test) from 297.48±47.57 m to 400.52±48.41 m after 12 months of treatment with sacubitril/valsartan (P<0.05), i.e., there was an increase of 20%. In contrast, in the valsartan group, the distance increased by 11% (P<0.05).

## Discussion

In this outpatient study of participants with HFrEF, treatment with sacubitril/valsartan resulted in a reduction in NT-proBNP level after 12 months of therapy compared with valsartan alone. Also, there was a significant reduction in secondary echocardiographic endpoints, including effective regurgitation orifice area, end-diastolic and end-systolic left ventricular volumes, left atrial volume, and E/e' index in the sacubitril/valsartan group. There was also a significant increase in LV ejection fraction in the sacubitril/valsartan group and in the 6-minute walk test distance. The data obtained indicated improvement of cardiac remodeling in the sacubitril/valsartan group.

Mitral regurgitation is a volume overload lesion that over time causes LV dilatation and eccentric hypertrophy. These size changes and LV remodeling is accompanied by changes in diastolic and systolic functions. SMR, which alters normal blood flow to and from the heart, may also increase LV end-diastolic volume (14). Adaptation to an incompetent left valve and its consequences underlie the associated morbidity and mortality. Typically, an adaptive response is viewed in terms of how it affects the size, remodeling, and function of the left ventricle. In patients with SMR, compensatory mechanisms that change the contractile ability to maintain hemodynamics in the face of increased afterload or preload may stop working over time, which may precede or accompany clinical deterioration of the patient's condition. The heart may undergo hypertrophic remodeling in response to a variety of stimuli (15). Depending on the stimulus, this hypertrophic growth may be partially or completely reversible. Numerous factors influence the type and extent of remodeling that may occur in a given individual, including age, sex, genetics, metabolic factors, coronary heart disease, and blood pressure (16). Overpressure, volume overload, or both due to valvular heart disease, may have an important effect on hypertrophic LV remodeling.

NT-proBNP is secreted primarily by the ventricular myocardium in response to increased wall stress with increased LV filling pressure (e.g., when expanding the volume of the ventricle or pressure overload) (17). Elevated serum NT-proBNP level is a predictor for mortality and hospitalization in patients with heart failure and coronary artery diseases or other cardiovascular diseases, as well as in SMR (18). It should be noted that even one assessment of the NT-proBNP level in the blood significantly facilitates risk assessment in patients with moderately to severely affected valves, in addition to echocardiography in outpatient settings (in addition to assessing effective regurgitation orifice area). Serum NT-proBNP should be considered as a cornerstone to evaluate and monitor patients with SMR for timely intervention.

Surgical correction of SMR is in great demand and includes endovascular treatment, mitral valve plastic surgery or prosthetics, LV plastic surgery, LV mechanical aid devices, and coronary artery bypass grafting for ischemic MR. However, given the etiological factors and despite the surgical treatment of MR, the outcome of the disease may be unfavorable (19). In our study, we assessed the effect of sacubitril/valsartan on hypertrophic LV remodeling and saw positive changes of NT-proBNP over time compared with valsartan alone. In the PARADIGM-HF study, sacubitril/valsartan also significantly reduced morbidity and mortality compared to enalapril (20) because enalapril alone, like valsartan, could not reverse unfavorable LV remodeling and SMR. Also, in the PIONEER-HF study in patients with HFrEF who were hospitalized for acute decompensated heart failure, the initiation of sacubitril-valsartan therapy led to a greater reduction in the NT-proBNP concentration than enalapril therapy (19). Another study noted that sacubitril/valsartan in patients with CHF and atrial fibrillation leads to a significant decrease in the level of brain natriuretic peptide and showed a clear trend towards reversing myocardial remodeling (21).

In addition to cardiac biomarkers, echocardiography provides a significant contribution to the evaluation of patients with HF and SMR. Changes in the level of NT-proBNP in our study correlated with greater changes in the effective area of the regurgitation orifice (as a criterion for assessing mitral regurgitation), LVEF, and size of the LV and left atrium of the group taking sacubitril/valsartan compared to the valsartan group. An increase in EF when taking sacubitril/valsartan was also found in other studies (22), as was improvement over 12 months of echocardiographic parameters such as index of left atrial volume, E/e', and indices of end-systolic and end-diastolic volume of the left ventricle (13). Specifically, there were no discrepancies in secondary echocardiographic points between the PRIME study (Pharmacological reduction of functional, ischemic mitral regurgitation) (23) and our study.

### Study limitations

The study population included the minimum number of participants. Another limitation of this study was the lack of patient randomization. For treatment, larger studies including randomized ones are needed. It is known that echocardiography is the standard imaging modality for evaluating mitral regurgitation, especially in outpatient settings, but echocardiography is not as accurate as magnetic resonance imaging in terms of measuring heart parameters and LV volumes.

### Conclusion

Among patients with SMR and HFrEF, treatment with sacubitril/valsartan resulted in a significant improvement in cardiac remodeling compared with valsartan alone.
